# A Review of the Carbon-Based Solid Transducing Layer for Ion-Selective Electrodes

**DOI:** 10.3390/molecules28145503

**Published:** 2023-07-19

**Authors:** Peike Wang, Haipeng Liu, Shiqiang Zhou, Lina Chen, Suzhu Yu, Jun Wei

**Affiliations:** 1 Shenzhen Key Laboratory of Flexible Printed Electronics Technology, Harbin Institute of Technology, Shenzhen 518055, China; 2 School of Materials Science and Engineering, Harbin Institute of Technology, Shenzhen 518055, China; 3 State Key Laboratory of Advanced Welding and Joining, Harbin Institute of Technology, Harbin 150001, China

**Keywords:** solid transducing layer, solid-contact ion-selective electrodes, carbon materials

## Abstract

As one of the key components of solid-contact ion-selective electrodes (SC-ISEs), the SC layer plays a crucial role in electrode performance. Carbon materials, known for their efficient ion–electron signal conversion, chemical stability, and low cost, are considered ideal materials for solid-state transducing layers. In this review, the application of different types of carbon materials in SC-ISEs (from 2007 to 2023) has been comprehensively summarized and discussed. Representative carbon-based materials for the fabrication of SC-ISEs have been systematically outlined, and the influence of the structural characteristics of carbon materials on achieving excellent performance has been emphasized. Finally, the persistent challenges and potential opportunities are also highlighted and discussed, aiming to inspire the design and fabrication of next-generation SC-ISEs with multifunctional composite carbon materials in the future.

## 1. Introduction

**Figure 1:**
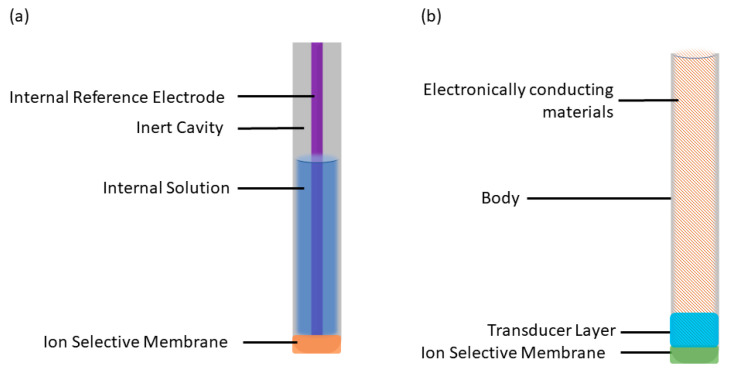
Structure images of: (**a**) LC-ISEs; (**b**) SC-ISEs.

The ion-selective electrode (ISE) is an electrochemical sensor that converts the activity of a target ion into a measurable electromotive force (EMF), enabling the determination of ion concentration. The first study of ISEs originated with Cremer’s discovery in the early 20th century, wherein variations in potential across a glass membrane reflected differences in H^+^ activity [1]. This finding sparked extensive and profound investigations into ISEs. The glass electrode, introduced in the 1930s, marked a significant milestone and triggered widespread exploration. During the 1960s and 1970s, remarkable advancements were achieved, including the development of the silver membrane ISE [2], the selective response of zinc oxide to combustible gases [3], and the advent of modern carrier-based ISEs. These liquid-contact ISEs (LC-ISEs) typically comprise an ion-selective membrane [4], an internal solution [5], a reference electrode [6], and an inert cavity [7] (Figure 1a). In the 1990s, Pretsch et al. [8] proposed the theory of steady-state ion flux in liquid ISEs, propelling a significant leap forward in understanding ISE mechanisms.

Today, liquid-contact ISEs are relatively mature and extensively employed. However, traditional LC-ISEs have their inherent limitations: (1) The evaporation and permeation of internal filling solution [9] and variations in sample temperature and pressure can affect the electrode response. (2) Osmotic pressure resulting from differences in ion strength between the sample and internal filling solution can cause the ingress of pure water into the internal filling solution, leading to significant volume changes or stratification of the ISM (internal solid membrane). This requires careful use and proper maintenance of ISEs, resulting in higher costs [10]. (3) There is a steady-state ion flux between the internal filling solution and the test solution. (4) It is challenging to reduce the volume of the internal filling solution to the milliliter level, making it difficult to miniaturize the electrode. To address these limitations, transitioning from the liquid interface to solid-state or solid-contact ISEs has emerged as a major research trend. SC-ISEs offer numerous advantages, including ease of storage, simplified maintenance, absence of external pressure requirements, low detection limits, reduced temperature dependence, and the potential for microfabrication [11]. Consequently, SC-ISEs have become a crucial research direction in the field of ISEs.

During the early development of SC-ISEs, the primary focus was on eliminating the need for filling solutions. In 1970, Hirata and Date et al. produced an all-solid-state Cu^2+^ ISE by coating platinum wire with CuS silica gel [12]. Subsequently, James et al. [13] conducted further research and coated a polymer-sensitive film containing a Ca^2+^ carrier onto a platinum wire, creating the first ion carrier-coated wire electrode. This resulted in the formation of a Ca^2+^ ISE, which is considered the first ion carrier-based SC-ISE. However, the coated electrode exhibited drawbacks such as poor stability and significant potential drift [13]. These issues arose from the absence of a stable ion–electron conversion between the sensitive film and the conductive substrate, as well as the small contact area of the wire-coated electrode, leading to noise and potential drift [14]. To overcome these challenges, it became necessary to introduce a solid contact material to facilitate the ion–electron conversion between the ion-selective membrane (ISM) and the conductive substrate. In 1985, Nikolskii and Materova [15] identified three key conditions for the solid contact (SC) layer to achieve stable and reliable SC-ISE responses: A reversible ion-to-electron conduction transition, an ideal non-polarized high exchange current density interface, and the absence of side reactions. Various SC materials have since been developed, including hydrogels [16,17], redox polymers [18,19,20], and self-assembled monolayers [21,22,23]. Hydrogel-based SC-ISEs, in particular, have gained significant attention. However, hydrogel-based electrolyte contacts do not completely eliminate the need for a filling solution but rather increase the sensor’s storage capacity under dry conditions. Moreover, the volume changes of hydrogels due to water adsorption/desorption are influenced by the ion concentration within the hydrogels.

In recent years, the field of SC-ISEs has witnessed significant advancements, driven by the need for improved stability, enhanced sensitivity, and the elimination of filling solutions. Using carbon materials as solid contacts in SC-ISEs is a promising area of research. Carbon materials, including carbon nanotubes (CNT) [24,25,26], graphene [27], carbon black (CB) [28,29], porous carbon, and other carbon materials [30,31,32] renowned for their unique properties, have emerged as highly suitable candidates for facilitating ion–electron conversion and addressing the limitations associated with traditional solid-contact materials. The incorporation of carbon-based solid contacts offers numerous benefits, including improved stability, reduced potential drift, hydrophobicity, and compatibility with various ion-selective membranes [33]. However, there is no systematic summary or analysis of the features of one specific carbon material that has been used for SC-ISEs. This paper has provided a comprehensive overview of the developments in the field of different carbon material-based SC-ISEs. The main objective of this research is to address the limitations associated with traditional solid-contact materials in SC-ISEs and explore the specific characteristics of the four main kinds of carbon materials (CNT, graphene, CB, porous carbon, and carbon-based composites) that have been researched for SCs. It is expected to offer more references for the selection of carbon materials as potential solid contacts to enhance stability, sensitivity, and wearability.

## 2. Carbon Materials for SC-ISEs

**Figure 2:**
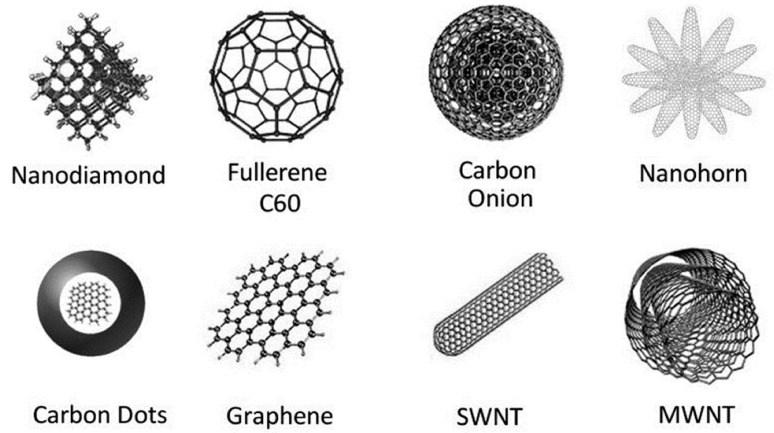
Members of the carbon nanomaterial family [34].

Carbon materials, known for their versatility and exceptional properties, have gained significant attention in various scientific and technological fields. Besides, carbon nanomaterials demonstrate structural diversity and often exist in the form of allotropes, including diamond, graphene, amorphous carbon, fullerene (C_60_), and carbon nanotubes (CNTs) (Figure 2) [34]. Moreover, they can also be classified based on spatial dimensions, such as zero-dimensional nanoparticles, one-dimensional carbon nanotubes, and two-dimensional layered materials such as graphene [35,36]. Over the past decade, carbon materials have gained attention as composite material carriers due to the following advantages: (1) Excellent acid and alkali resistance; (2) structural stability at high temperatures; (3) high chemical and mechanical reliability; (4) adjustable pore and surface properties; (5) controllable active sites; (6) good electrical and thermal conductivity; (7) versatile structures and morphologies for ease of manipulation; and (8) low production costs [37,38]. These materials exhibit unique properties in terms of size, surface area, strength, and electrical properties. They also possess chemical diversity, ease of manipulation, biocompatibility, and stability as carrier materials. Therefore, they have become attractive research subjects in various fields such as catalysis, energy storage, adsorption, and sensing.

Carbon-based SCs bring several benefits to ISEs, including enhanced stability, sensitivity, selectivity, and hydrophobicity. The incorporation of carbon materials in SC-ISEs facilitates efficient ion–electron conversion, enhances electrode–substrate interactions, and provides a stable and reliable sensing interface. Moreover, carbon materials enable miniaturization, flexibility, and compatibility with various substrates, expanding the possibilities for the development of wearable and portable SC-ISEs. The comparison of performance across typical carbon-based SC-ISEs is shown in Table 1.

### 2.1. Carbon Nanotubes (CNTs)

**Figure 3:**
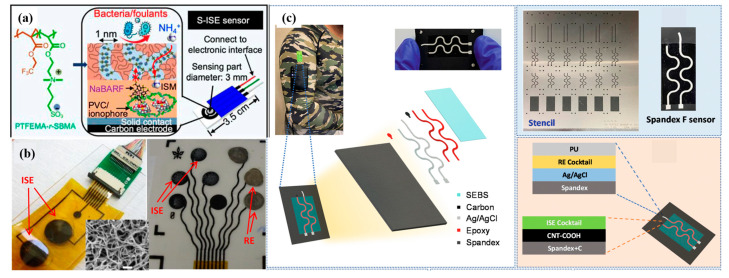
(**a**) Chemical structure of self-assembled channel-type zwitterionic copolymer poly (trifluoromethyl methacrylate-random-sulfobetaine methacrylate) PTFEMA-r-SBMA (**left**); self-assembly of PTFEMA-r-SBMA from the network of hydrophilic nanochannels ∼1 nm in diameter for anti-biofouling and leaching prevention properties (**middle**) and diagram of S-ISE sensors (**right**) [51]. (**b**) CNT-based flexible ISEs, which contained ISE and reference electrode [39]. Fabrication flow of CNT electrodes. And ISEs on polyimide (Kapton) substrates. (inset) SEM image of the surface of the CNT electrodes. (**c**) Flexible and stretchable textile-based solid-contact potentiometric fluoride sensor [53].

CNTs are cylindrical structures composed of rolled graphene sheets, offering exceptional mechanical strength, high electrical conductivity, and a large surface area. These properties make CNTs an excellent choice for SC-ISEs. The use of CNTs as solid contacts improves ion–electron transfer, reduces potential drift, and enhances the overall stability and sensitivity of the electrodes [46,47,48]. Rius et al. [26,49,50] first used a single-walled carbon nanotube (SWCNT) as a solid-state transfer layer to stabilize electrode potential because of its large capacitance property and then used CNT instead of an ion-selective membrane in the following research. It is used in the detection of biological macromolecules, proteins, etc. Then, to prove the flexibility and wearability, Huang et al. [51] successfully added a biological antifouling film to SWCNT SCs (Figure 3a). This ISE can be used for long-term, continuous monitoring of water quality. This research confirmed the role of SWCNT in reducing water layer formation and reinforcing the ISM and electrode link.

Another kind of material used as SCs is multi-walled CNT (MWCNT). In 2017, a remarkable advancement was presented. Roy et al. [39] produced a wearable sensor constructed using multi-walled CNT (MWCT) electrode arrays for sweat sensing. SC-ISEs with a specific sensitivity to Na^+^ ions were fabricated by drop coating plasticized poly (vinyl chloride) (PVC) doped with ionophore and ion exchanger onto CNT electrodes (Figure 3b). The ion-selective membrane (ISM) effectively filled the intratubular spaces within the highly porous CNT film, resulting in a stronger attachment compared to flat Au, Pt, or carbon electrodes. Remarkably, a sensitivity of 56 ± 3 mV/decade to Na^+^ ions was achieved. This study successfully demonstrates the immense potential of CNT-based devices integrated onto flexible supports as an enticing platform for future wearable technology devices. Then Wang et al. [52] proposed a smooth elastic fiber (SE-fiber) coated with CNT film. The effective combination of the wrinkle conductive electrode with the “island” region-designed ion-selective electrode and reference electrode could lead to a highly stable sensing performance, ignoring the stretching/releasing of the sensor from 0 to 200% strain. However, upon releasing the prestretched SE fiber, the CNT film could form a conformal wrinkle structure along with the substrate. In contrast, the sensing materials, including the Ag/AgCl ink film and ion-selective membrane, gradually detached from the CNT layer of the fiber. In order to fabricate a more stable electrochemical sensor on a highly stretchable elastic fiber based on wrinkle structures, carboxyl-functionalized multi-walled carbon nanotube (MWCNT) SCs were also used to make the first wearable, textile-based solid-state contact fluoride sensor (Figure 3c) [53].

### 2.2. Graphene

Graphene is a two-dimensional sheet of carbon atoms arranged in a hexagonal lattice, exhibiting extraordinary electrical, mechanical, and thermal properties. Its high electrical conductivity and large surface area make graphene an ideal material for SC-ISEs. Graphene-based SC-ISEs offer enhanced sensitivity, selectivity, and stability. The carbon atom in graphene is bonded to three other carbon atoms; however, the valency of carbon is four; hence, one of the electrons is free to move around in the sheet, leading to an exceptionally high carrier concentration in graphene [54]. The synthesis method used to prepare graphene is crucial since the defects in graphene, such as corrugations (wrinkles, ripples, or crumples), dislocations, adatoms, and Stone–Wales defects, determine its electrical behavior.

**Figure 4:**
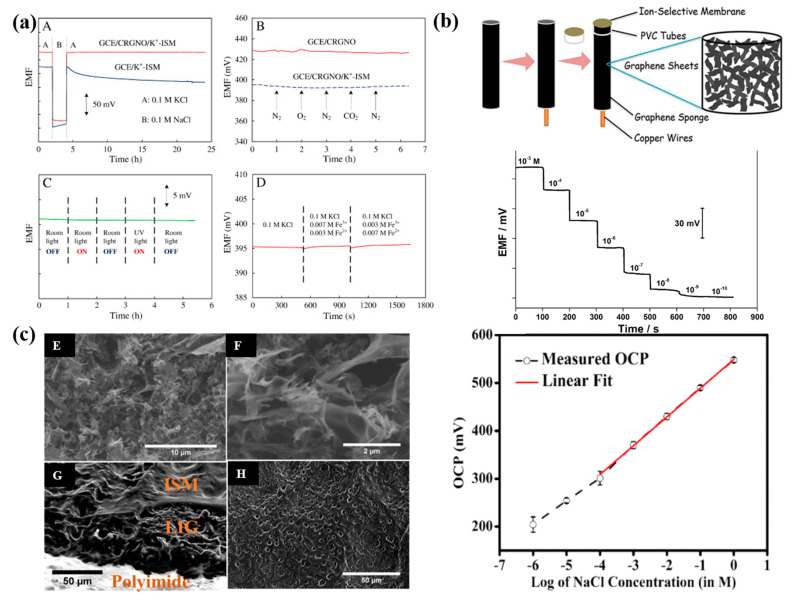
(**a**) Stability test for the GCE/K^+^-ISM and GCE/CRGNO/K^+^-ISM in different environments [55]. (**A**): Water layer test for the GCE/K^+^-ISM and GCE/CRGNO/K^+^-ISM, the measurements were switched between 0.1 M KCl and 0.1 M NaCl. (**B**): Sensitivity to O_2_ and CO_2_ in 0.1 M KCl solution for the GCE/CRGNO (solid line) and GCE/CRGNO/K^+^-ISM (dashed line). (**C**): Effect of light on the potential stability of the GCE/CRGNO/K^+^-ISM in 0.1 M KCl solution. (**D**): Redox interference test for the GCE/CRGNO/K^+^-ISM, the solution was switched to the mixed solution containing 0.1 M KCl, 0.007 M FeCl_3_ and 0.003 M FeCl_2_ at 500 s, then replaced by the mixed solution containing 0.1 M KCl, 0.003 M FeCl_3_ and 0.007 M FeCl_2_ at 1000 s. (**b**) A freestanding all-solid-state polymeric membrane Cu^2+^-selective electrode based on three-dimensional graphene sponge was fabricated, and time-dependent responses of the GS/Cu^2+^-ISE to Cu^2+^ in the activity range of 7.9 × 10^−4^–1.0 × 10^−10^ M [56]. (**c**) Potentiometric ISEs based on UV-ozone-irradiated laser-induced graphene electrode [40]. (**E**,**F**): SEM images Ozone treated LIG. (**G**): Cross-sectional SEM image of ISE prepared by two-step drop-coating process showing the different layers. (**H**): SEM image showing the porous morphology of the top surface of an ISE.

In 2011, graphene was first used as a solid ion-to-electron transducer in ISEs [55]. This novel electrode displayed a Nernstian response of 58.4 mV/decade and an impressively low detection limit of 10^−6.2^ M. Importantly, the potentiometric water layer test confirmed the absence of water film formation between the solid-contact layer and the polymeric membrane (Figure 4a). Furthermore, the developed electrode exhibited a fast response, was insensitive to oxygen and light, and had excellent potential stability, which makes it very promising for routine analysis and application (Figure 4a). The 3D graphene sponge (3D GS) was proven to be used as both an electrode substrate and a solid contact for the construction of Cu^2+^-ISE [56]. This electrode shows a stable potential response with a LOD of 2.5 × 10^−9^ M (Figure 4b). Then, the laser-induced graphene (LIG) technique [57], three-dimensional (3D) self-assembled porous graphene aerogel (PGA) [58], and cold atmospheric plasma surface modification [59] were also successfully electrodeposited on the electrode substrate surface. What needs to be mentioned is that ultra-thin and defect-free graphene ink was applied in the preparation of the screen-printed sensor, which significantly enhances the mechanical flexibility of ISEs [60]. On the other hand, Sudiptad et al. used ultraviolet-ozone irradiation to induce laser-induced graphene (LIG) electrodes and developed a disposable device for detecting biosignals such as Na^+^ ions in sweat [40]. The resulting device exhibited a sensitivity of 60.2 ± 0.9 mV/decade towards Na^+^. The stability characteristics and significantly expanded preparation methods for graphene-based SCs make it theoretically possible to produce wearable SC-ISEs on a large scale and finally achieve commercial application (Figure 4c).

### 2.3. Carbon Black (CB)

CB, which consists of fine particles of amorphous carbon, is widely used in several industrial processes. This nanomaterial features a set of remarkable properties, including high surface area, high thermal and electrical conductivity, and very low cost [61]. Several studies have explored the applicability of CB in electrochemical fields. Carbon black-based SC-ISEs have also been employed for the detection of various analytes due to their improved sensitivity and stability [62]. The porous nature of carbon black allows for increased ion diffusion and enhanced electrochemical performance. Previous studies show that CB has the ability to intercalate ions on graphite layers [63], which affects electrical conduction between particles by tunneling [64] and the surface reaction. These characteristics also make CB a valuable material for SC-ISEs.

**Figure 5:**
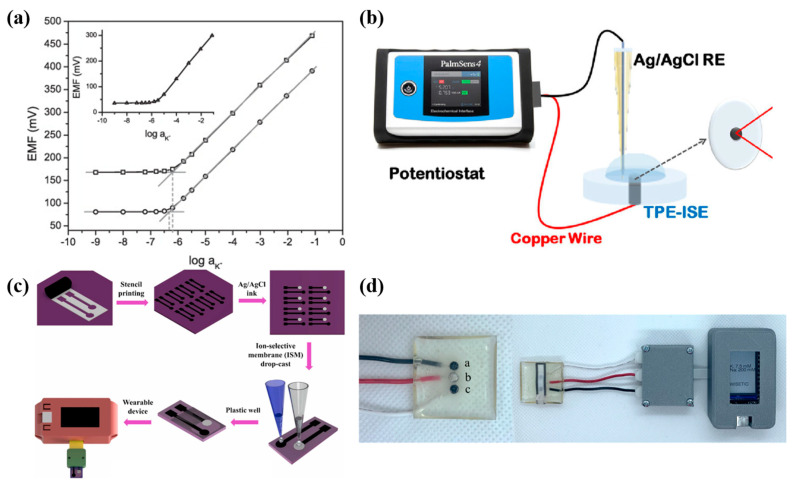
(**a**) EMF dependence on K^+^ activities for GCD/CB/K^+^-ISM (○), GCD/(CB, K^+^-ISM) (□), and inset: GCD/K^+^-ISM (△) electrodes conditioned in 0.01 M KCl solution [65]. (**b**) CB-modified K^+^ sensitive thermoplastic electrode [66]. (**c**) Fabrication process of K^+^-selective SPCEs and integration into the wearable device (WISETIC) [68]. (**d**) Image of the TPE array, where a is the Na^+^-ISE, b is the reference electrode, and c is the K^+^-ISE, and the TPE array is coupled to a paper-based device (PAD) [69].

In 2012, Paczosa-Bator successfully introduced carbon black into SC-ISE for the first time [65]. CB was functional as an intermediate layer between an ionophore-doped solvent polymeric membrane and an electrical conductor and a polymeric membrane component, respectively. As shown in Figure 5a, the developed electrodes show a very stable response over time. Even after a long time of conditioning in 0.01 M KCl (6–7 weeks), the electrodes still showed a linear response in the same range of potassium activity. Notably, in 2022, all-solid-state K^+^-selective sensors based on CB-modified thermoplastic electrodes were fabricated [66]. The result in Figure 5b shows that modification of CB can reduce noise and the formation of a water layer between graphene solid contact and polymeric membrane due to the small particle size of graphene and the CB hydrophobicity, which contributes a lot to the stability of SC-ISEs.

All these studies prove that CBs in SC-ISE can improve the long-term stability of the devices. However, compared to CNT and graphene, CB SC-ISEs have a much higher low detection limit (around 10^−4^~1 M [28]). And compared with SCs, CBs are more suitable for electrode modification. Due to the high capacitance, CB can efficiently reduce the resistance and mediate the charge transfer at the ISM and glassy carbon electrode interface [67]. Ozer et al. achieved a good sensitivity response to K^+^ and a LOD of 1 × 10^−5^ M after modifying stencil-printed carbon electrodes (SPCE) [68] and microfluidic paper-based thermoplastic electrodes (TPE) [69] with CB, respectively (Figure 5c,d).

### 2.4. Porous Carbon

Porous carbon materials have high electrical conductivity and a large specific surface area [70], and they also have an adjustable pore size, which improves the selectivity of the ISE in the detection process [71]. Among all, three-dimensionally ordered microporous carbon (3DOMC) draws researchers’ attention. It consists of a glassy carbon skeleton with interconnected macropores that can be infiltrated with the ISM to form a continuous structure in which electrons are conducted through the carbon framework while ions move through the infused ISM. Its large interfacial contact area and high capacitance mean that it has a larger contact area and higher EDL capacitance when used as SCs, which is more conducive to electron transfer and potential stability.

**Figure 6:**
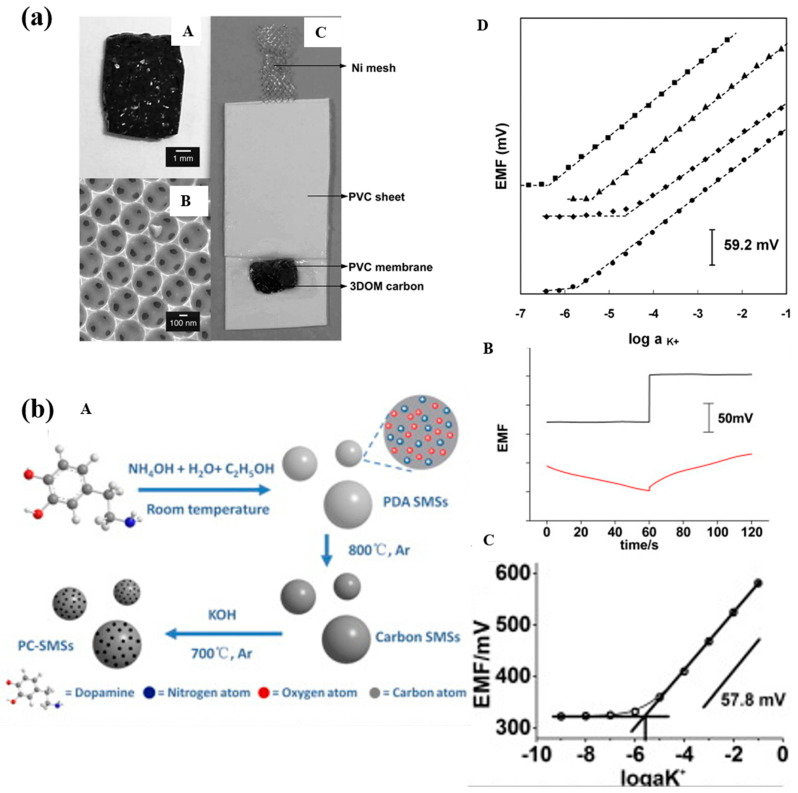
(**a**)—(**A**): Photograph of monolithic 3DOM carbon. (**B**): SEM image of 3DOM carbon. (**C**): Photograph of 3DOM carbon-contacted ISE. (**D**): Potentiometric K^+^ response curves of SC-ISEs with different electrode assemblies in KCl solutions: (◼) Ni/3DOM carbon/PVC, (▲) Ni/HOPG/PVC, (◆) Ni/3DOM carbon, (●) Ni/PVC. For clarity, response curves have been shifted vertically relative to one another [41]. (**b**)—(**A**): Schematic illustration of the synthesis of PC-SMSs. (**B**): Chronopotentiometric results. Upper part: PC-SMSs/K^+^-ISE; applied current: 10 nA for 60 s and −10 nA for 60 s; bottom part: K^+^-CWE; applied current: 1 nA for 60 s and −1 nA for 60 s; solution: 0.1 M KCl. (**C**): Calibration curve for the PC-SMSs/K^+^-ISE in the varied concentration of KCl solution [31].

Lai et al. [41] first employed 3DOMC as a solid-state transduction layer for K^+^-selective electrodes, as shown in Figure 6a. The SC-ISEs fabricated based on 3DOMC exhibited a long-term potential drift of only 11.7 mV/h, which remained unchanged over an extended period. The electrode demonstrated excellent resistance to interference from oxygen. Additionally, the team investigated the influence of 3DOMC structure and surface chemistry on the performance of ion-selective electrodes. It was shown that oxidized 3DOMC exhibited a typical water layer phenomenon that led to the potential stability of the deterioration of SC-ISEs [72]. In addition, Niu’s team also developed porous carbon submicrons (PC-SMSs) as SCs for K^+^ SC-ISE, as shown in Figure 6b. PC-SMSs have high hydrophobicity and a large EDL capacitance. The potential drift of PC-SMS-based ISEs is only 7 mV in two months [31].

### 2.5. Other Carbon Materials

**Figure 7:**
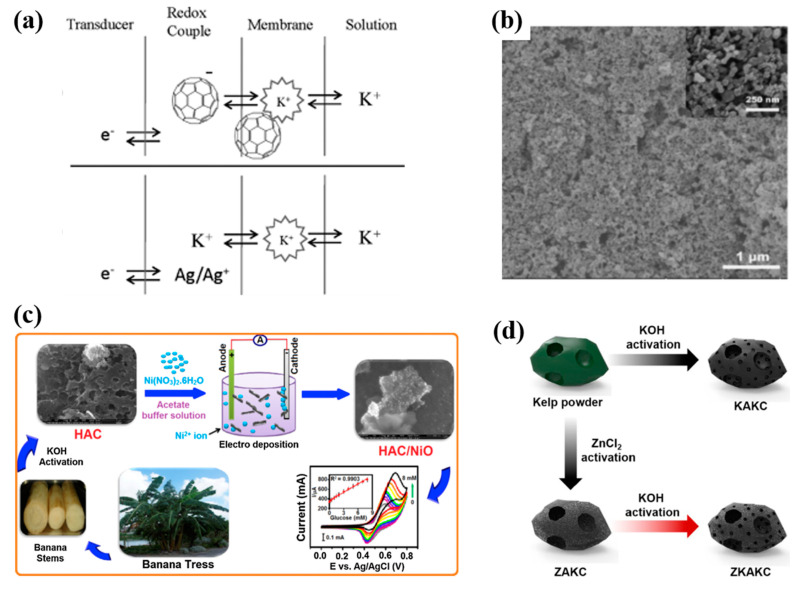
(**a**) Schematic diagram of the charge-transfer process in a C_60_ SC-ISEs (upper) and conventional ISEs (down) [42]. (**b**) SEM image of GCE/SWCNHs [43]. (**c**) Illustrated synthesis of HAC/NiO nanocomposite from banana stems, their application as glucose sensor and CV curves [44]. (**d**) Graphical illustration of preparation of ZKAKC [45].

Apart from the four main carbon materials, a wide variety of carbon materials are available. This makes it possible for the researchers to choose the most appropriate SC material to satisfy the specific requirements and desirable performances. For example, zero-dimensional hollow spherical fullerenes with polygons [42,73], single-walled carbon nanohorns (SWCNHS) [43], NiO-doped porous biomass carbon materials [44], ZnCl_2_-KOH-activated kelp charcoal (ZKAKC) [45], graphdiyne oxide [74], and other functionalized carbon composites [75,76,77,78] were also used as SC-ISEs of SCs and achieved good results (Figure 7).

**Figure 8:**
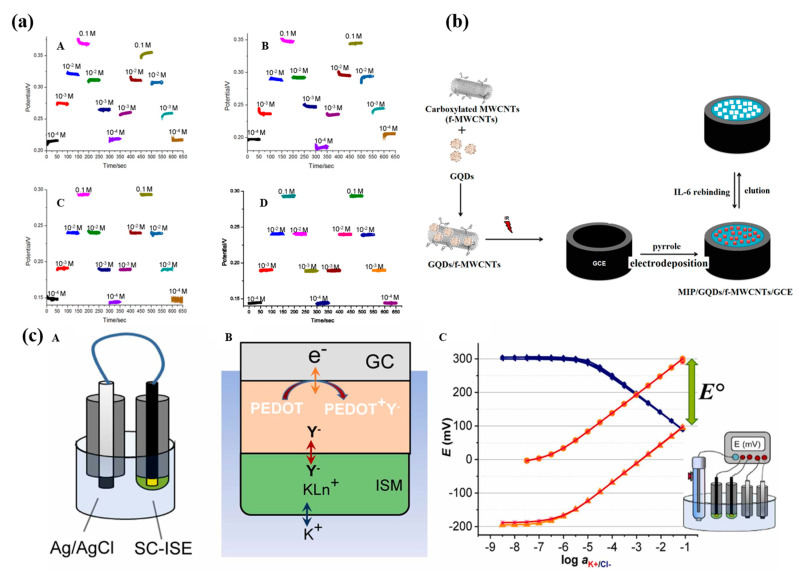
The reversibility of the potentiometric NH_4_^+^ detection using sensors (**a**)—(**A**,**B**):without and (**C**,**D**): with 3D graphene–CNT modified electrodes, respectively [79]. (**b**) Schematic preparation of molecularly imprinting polymers (MIP)/GQDs/f-MWCNTs/GCE [81]. (**c**) Schematic representation of the instrument-free method to adjust the standard potential E^0^ of solid-contact ion-selective electrodes. (**c**)—(**A**): The SC-ISE is short-circuited with an Ag/AgCl QRE in a solution of constant concentration of the chloride salt of the primary ion. (**B**): The potential difference between the short-circuited electrodes causes oxidation/reduction or charging/discharging of the solid contact (depending on the type of solid-contact material) which (**C**): Alters the standard potential of the short-circuited SC-ISEs measured at open circuit potential, while the potential of the Ag/AgCl remains constant due to its large capacitance [82].

At present, another trend of SCs is to combine carbon materials with various materials in order to take advantage of each other. Due to the hydrophobic properties of the 3D graphene oxide–CNT composite, Hua et al. successfully achieved higher reversibility of ISEs (Figure 8a). Under a strain of 40%, the sensor still showed a sensitivity of 42.7 ± 3.1 mV/log (NH_4_^+^) [79]. Another study also proved that the ionic liquid trihexyltetradecylphosphonium chloride (THTDPCl) and various types of multi-walled carbon nanotubes (MWCNTs-THTDPCl) nanocomposite effectively act as an ion exchanger and ionic membrane component by decreasing membrane resistance. Such a device does not require additional lipophilic salt [80].

In addition, the carbon material’s surface can also be functionalized with various functional groups (such as oxides, amides, thiols, or different groups) to change the properties of nanomaterials. This strategy can satisfy the special properties of ISEs, such as small quantities of samples, free of calibration, and long-term stability, making it possible to work in specific conditions. Nermin et al. used graphene quantum dots (GQD)/carboxylated CNT (f-CNT) as SCs and found that GQDs/f-MWCNTs can not only prevent the stacking of GQDs but also have a larger surface area (Figure 8b). Their hydrophilic groups also contribute to the reduction of sample quantities [81]. In another work, a larger capacitance quasi-reference electrode (QRE) was used to calibrate SC-ISEs (Figure 8c). Therefore, upon short-circuiting, the potential of the QRE remains practically constant, while the potential of the SC-ISE is shifted until the potential difference between the SC-ISE and QRE approaches zero [82].

## 3. Relationship between Carbon Materials and Performance of the SC-ISEs

According to the International Union of Pure and Applied Chemistry (IUPAC) on the definition of ISE-related terms, evaluation of performance indicators, and identification methods, the performance evaluation of ISEs includes sensitivity, selectivity, detection limit, responsible time, and service life. Among these, the sensitivity of ISEs is described by the Nernst equation. However, the diffusion potential of ions between samples depends mainly on the ISM. So, it will not be discussed in detail in this work.

1. Selectivity: Selectivity is one of the most important characteristics of the sensor because it determines whether the target ion can be reliably measured. In the actual measurement, ISEs will also respond to some interference ions, so that the response slope of the electrode deviates from the theoretical value and the sensitivity decreases. Therefore, it is necessary to introduce a selectivity coefficient to evaluate the anti-interference ability of electrodes against interfering ions. The smaller the selectivity coefficient $K_{A,B}^{pol}$, the better the ISE selectivity and the better the anti-jamming capability [83].
2. Detection range: Each ISE has an upper and lower detection limit, and the range between the upper and lower detection limits is the detection range of the electrode. With the decrease in the activity of the target ions, the interfering ions can enter the ISM, which results in a deviation of the response of the electrode from the theoretical value of the Nernst slope. And the lowest concentration of this solution is regarded as the lower limit of detection. When the target ion activity is too high, the ISM will produce a co-extraction effect with the solution to be measured, which also causes the response of the electrode to deviate from the theoretical value of the Nernst slope. It is defined as the upper limit of detection [84].
3. Response time: In continuous monitoring, rapid response is an important parameter to obtain the dynamic change of target ion activity in real time. The response time is considered to be the time required for the sensor to reach 90% of its equilibrium potential [85,86]. The main factors affecting the electrode response time are the diffusion-controlled equilibrium of target ions at the membrane-water interface and the efficiency of ion–electron conversion, which depend on the ISM and SC, respectively.
4. Lifetime: The service life of electrodes refers to the time that the electrode can be used normally while keeping its performance indexes unchanged. The service life of electrodes is mainly affected by the ISM, such as the aging of the membrane matrix, the loss of ionic carriers and additives, and the damage of the external environment to membrane components [87].

ISEs incorporate SCs to enable reversible ion–electron conversion and stabilize the SC|ISM interface potential. This maximizes the performance of SC-ISEs, aiming to create highly stable, maintenance-free, calibration-free, and interchangeable potential ion sensors. The exceptional performance of SC materials is crucial for ensuring stable and reproducible potential. Among these capabilities, a stable potential response is indicative of minimal potential drift, while poorer potential stability typically suggests defects in the SC material. Additionally, the reproducibility of potential among individual electrodes determines calibration frequency, while the reproducibility of electrode standard potential (E^0^) between electrodes determines their direct interchangeability. Therefore, when compared to LC-ISEs, the performance assessment of SC-ISEs includes evaluating potential stability, reproducibility, the presence of a water layer, and interference resistance [65].

1. Stability: Ideally, SC materials should possess a non-polarizing interface with a high exchange current density. However, in practical measurements, the input current of the measuring amplifier inevitably causes charging and discharging, resulting in varying degrees of electrode polarization [88]. In addition to electrode polarization, mechanical failures of the electrode and hydrolysis of the ISM can also lead to potential drift. For instance, when the electrode is immersed in an aqueous solution for an extended period, the adhesion between the ISM and the SC gradually decreases, resulting in potential fluctuations [89]. Extensive theoretical and practical evidence demonstrates that a sufficiently large oxidation–reduction or electric double-layer (EDL) capacitance serves as a guarantee for electrode potential stability [90]. In the EDL model, the sum of the three interface potentials (ISM|(SC)|GC) is the total potential of SC-ISEs. However, due to the conductivity of SCs, the potential diversity of SC|GS can be ignored. And the potential diversity between SCs and ISMs cannot be accurately calculated because there is no electron exchange between SCs and ISMs. But according to the definition of potential (E = Q/C, E represents potential diversity, Q represents the amount of charge, and C represents capacity), it is easy to realize that a larger capacity of SCs would result in a smaller potential diversity of SC|ISM. Most carbon materials used for SCs belong to the capacitance-based transduction mechanism. It means carbon materials with a large surface area and abundant pores, such as graphene, SWCNT, and porous carbons, are advantageous for increasing capacitance and enhancing the stability of ISEs. At the same time, a hydrophobic surface can also help reduce drift currents.
2. Reproducibility: The pre-calibration of potential is an essential step for SC-ISEs before testing, as it directly affects the accuracy and reproducibility of the measurement results. While SC-ISEs with low potential drift can meet the requirements of practical testing through periodic calibration, complex or frequent calibrations can significantly increase time and cost [87]. Achieving high reproducibility for the E^0^ remains a challenge. The Bühlmann group has pointed out that E^0^ is determined by the overall structure of SC-ISEs, including each bulk phase and interface [91]. It is very important to reduce the surface redox functional groups to maintain the stability of the interface potential. When it comes to carbon materials, The introduction of colloidal imprinted mesoporous carbon (CIMC) can effectively improve the reproducibility of the E^0^ [92]. Besides, redox buffer has already been introduced to design and fabricate calibration-free ion sensors [93], but their lifetime is not long because of the gradual loss of the redox buffer from the ISMs with usage time. In summary, research on E^0^ is still in its early stages, and there is room for improvement in terms of potential reproducibility. The development of calibration-free SC-ISEs remains a hot topic for future research.
3. Water layer testing: The water layer effect at the SC|ISM interface is one of the major and persistent challenges in SC-ISEs (solid contact ion-selective electrodes). The continuous water layer formed between SCs and ISMs acts as a reservoir for transmembrane ions and neutral particles, but instead of providing a reversible interfacial potential, it irreversibly disrupts the long-term potential stability of SC-ISEs. As the water layer continues to diffuse at the interface, the adhesion between ISMs and SCs is further compromised, eventually leading to the separation of ISMs from the substrate [94]. This is also a reason that causes a change in the value of E^0^ [95]. Currently, enhancing the hydrophobicity of SC materials is considered the most effective approach to addressing the water layer effect [96]. Most carbon materials possess a high surface area and strong adsorption capabilities due to their porous nature. Therefore, methods to enhance the hydrophobicity of carbon materials include reducing the presence of oxygen-containing hydrophilic functional groups on the surface and defects.
4. Interference testing: In addition to the water layer effect, various external interferences such as light, CO_2_, O_2_, and redox couples can also disrupt the ion–electron transduction process and lead to potential changes. This necessitates that SC functional materials possess sufficient capacitance and high chemical stability. Actually, there are four electrons in the outermost layer of carbon. These four electrons occupy the s and p sublayers of the second layer. There are four total orbitals in these two layers. According to the Hund rule, each electron occupies one orbital and chooses the same direction; the electrons are low in energy and relatively stable, which is why the carbon chemical property is so stable. In this regard, most carbon materials are relatively hard to react with disturbing effects and meet the requirements of SCs.

## 4. Conclusions and Outlook

Through the development and research process of ISEs, in order to address the issues of calibration, large volume, leakage, and short lifespan associated with LC-ISEs, researchers introduced the concept of SCs. This was performed with the aim of developing ISEs that exhibit strong stability, can adapt to complex testing environments, and facilitate miniaturization. However, in order to achieve stable and reliable responses in SC-ISEs, SCs need to meet certain criteria, including reversible ion-to-electron conduction, an ideal non-polarizing high exchange current density interface, and no side reactions. Various materials, including noble metals, conductive polymers, and carbon materials, have been studied as SCs. Among these materials, carbon materials have gained wide applications due to their abundance, good conductivity, and low cost. However, some current challenges and future directions for carbon materials in SC-ISEs are as follows:

1. Different types of carbon-based SCs have their own advantages. CNT-SCs exhibit higher capacitance. Graphene SCs have good resistance to interference and are applicable to various preparation methods. CB-SCs demonstrate better repeatability and long-term stability but have a narrower measurement range. Porous carbon SCs exhibit stable calibration potential and hydrophobicity.
2. Limited selectivity: The carbon-based solid transducing layer may exhibit lower selectivity towards certain ions, leading to cross-interference or inaccuracies in ISE measurements. This can restrict the accuracy and precision of ion analysis. Developing carbon-based solid transducing layers with improved selectivity towards specific ions is an important research direction. To enhance ion recognition and selectivity, the design and modification of carbon materials, such as introducing functional groups or surface modifications, are effective ways.
3. Surface adsorption and contamination: Carbon materials have a high adsorption capacity, making them prone to the adsorption of impurity ions or organic substances. This can result in surface adsorption and contamination and affect the selectivity and stability of ISE. In terms of surface design, surface modification of SCs by adding redox buffers, hydrophobic layers, and other methods is used to achieve stable E^0^ and drift potential. Investigating surface engineering strategies is essential to reducing adsorption and fouling on the carbon-based solid transducing layers. Besides, surface modifications, coatings, and nanostructured surfaces are all efficient in minimizing interference from impurities and enhancing the stability and performance of SCs.
4. Structural degradation and deterioration: Prolonged usage or cycling processes may lead to structural changes or degradation of the carbon-based SCs and cause performance deterioration or irreversible damage. It would also limit the long-term stability and reliability of ISEs. Exploring the integration of carbon-based SCs with other advanced technologies, such as nanomaterials, nanoelectronics, or microfluidics, can unlock new possibilities for enhanced and multifunctional ISEs. Such a way includes exploring composite materials, hybrid structures, and novel device architectures that leverage the unique properties of carbon materials.

## Figures and Tables

**Table 1:** Comparison of performance across typical carbon-based SC-ISEs.

SC Materials	Base Electrode	Target Ion	Sensitivity (mV/Decade)	Range (M)	Response Time (s)	Ref
CNT	CNT	Na^+^	56 ± 3	7.08 × 10^–7^ to 1	57	[39]
Graphene	Carbon electrode	Na^+^	60.2 ± 0.9	1 × 10^−6^ to 1	60	[40]
CB	SPE	Na^+^	58 ± 3	1 × 10^−7^ to 1		[28]
3DOMC	Ni mesh	K^+^	56.4	1.6 × 10^−7^ to 1		[41]
PC-SMSs	GCE	K^+^	57.8	1 × 10^−6^ to 1		[31]
Fullerenes	GCE	K^+^	55			[42]
SWCNHS	GCE	Ca^2+^	27.14	1 × 10^−6.1^ to 1 × 10^−2^	4	[43]
HAC/NiO	GCE	Glucose		5.5 × 10^−8^ to 1		[44]
ZKAKC	GCE	Acetaminophen		1 × 10^−8^ to 2 × 10^−5^		[45]

CNT: carbon nanotube; CB: carbon black; 3DOMC: three-dimensionally ordered microporous carbon; GCE: glassy carbon electrode; HAC: heteroatom-enriched porous carbon; PC-SMSs: porous carbon submicrons; SPE: screen-printed electrode; SWCNHS: single-walled carbon nanohorns; ZKAKC: ZnCl_2_-KOH-activated kelp charcoal.

## Data Availability

No new data were created or analyzed in this study. Data sharing is not applicable to this article.
